# Supplementary material for: Transcriptomic Analysis of Hub Genes Reveals Associated Inflammatory Pathways in Estrogen-Dependent Gynecological Diseases
Source: Biology (Basel). 2024 May 30;13(6):397. doi: 10.3390/biology13060397 (PMC11201105; doi:10.3390/biology13060397)
Supplement: Supplementary file 1 [file biology-13-00397-s001.zip › Supplementary Materials.pdf]

Transcriptomic Analysis of Hub Genes Reveals Associated Inflammatory Pathways in Estrogen-Dependent Gynecological Diseases

Elaine C. Pasamba, Marco A. Orda, Brian Harvey Avanceña Villanueva, Po-Wei Tsai and Lemmuel L. Tayo

Table S1. Summary of hub genes within GO Terms and Pathways.

Module		KEGG Pathway	Count	*p*-Value	Hub genes
Cyan	hsa04810	Regulation of actin cytoskeleton	10	3.75E-03	KRAS, SOS1, PTK2
	hsa05208	Chemical carcinogenesis - reactive oxygen species	8	3.12E-02	KRAS, SOS1, PTK2
	hsa04660	T cell receptor signaling pathway	6	2.38E-02	KRAS, SOS1
	hsa05100	Bacterial invasion of epithelial cells	5	2.01E-02	PTK2
	hsa04012	ErbB signaling pathway	5	2.77E-02	KRAS, SOS1, PTK2
Midnightblue	hsa05168	Herpes simplex virus 1 infection	15	3.32E-02	CASP3
	hsa05010	Alzheimer disease	11	8.58E-02	CASP3
	hsa05160	Hepatitis C	7	4.03E-02	CASP3, CDK6
	hsa04614	Renin-angiotensin system	4	5.55E-03	ENPEP
Pink	hsa04110	Cell cycle	52	8.74E-35	CDK1, CCNB1, CCNA2, BUB1B, AURKB, CDC20, BUB1, CDC45
	hsa04218	Cellular senescence	20	5.72E-06	CDK1, CCNB1, CCNA2
	hsa05166	Human T-cell leukemia virus 1 infection	20	6.94E-04	CCNA2, BUB1B, CDC20, BUB1
	hsa05203	Viral carcinogenesis	15	2.18E-02	CDK1, CCNA2, CDC20
	hsa04115	p53 signaling pathway	12	9.06E-05	CDK1, CCNB1
Purple	hsa04371	Apelin signaling pathway	10	3.01E-02	KRAS
	hsa04926	Relaxin signaling pathway	9	4.91E-02	EGFR, KRAS
	hsa03083	Polycomb repressive complex	8	1.58E-02	
	hsa00520	Amino sugar and nucleotide sugar metabolism	6	1.79E-02	
	hsa05219	Bladder cancer	5	3.83E-02	TP53, EGFR, KRAS

Tan	hsa03040	Spliceosome	18	1.16E-04	FUS, RBM25, SNRNP70
	hsa05014	Amyotrophic lateral sclerosis	18	2.63E-02	HNRNPA2B1, FUS
	hsa05202	Transcriptional misregulation in cancer	12	1.92E-02	FUS
	hsa03015	mRNA surveillance pathway	9	5.63E-03	FUS
	hsa00310	Lysine degradation	6	3.09E-02	

Table S2. Summary of hub genes within highly preserved modules.

Module	Gene	Protein
Cyan, and Purple	KRAS	KRAS proto-oncogene, GTPase
Cyan	HNRNPA1	heterogeneous nuclear ribonucleoprotein A1
	SOS1	SOS Ras/Rac guanine nucleotide exchange factor 1
	YWHAZ	tyrosine 3-monooxygenase/tryptophan 5-monooxygenase activation protein zeta
	CALML6	calmodulin like 6
	AKAP6	A-kinase anchoring protein 6
	LARP7	La ribonucleoprotein domain family member 7
	PTK2	protein tyrosine kinase 2
	UBE2D3	ubiquitin conjugating enzyme E2 D3
	SMAD4	SMAD family member 4
Midnightblue	ESR1	estrogen receptor 1
	CASP3	caspase 3
	ITIH4	inter-alpha-trypsin inhibitor heavy chain family member 4
	SQSTM1	sequestosome 1
	NRXN1	neurexin 1
	ENPEP	glutamyl aminopeptidase
	CDK6	cyclin dependent kinase 6
	DISC1	disrupted in schizophrenia 1
	CD9	CD9 molecule
	GPX7	glutathione peroxidase 7
Pink	CDK1	cyclin dependent kinase 1
	CCNB1	cyclin B1
	TOP2A	topoisomerase (DNA) II alpha
	CCNA2	cyclin A2
	BUB1B	BUB1 mitotic checkpoint serine/threonine kinase B
	EXO1	exonuclease 1

	AURKB	aurora kinase B
	CDC20	cell division cycle 20
	BUB1	BUB1 mitotic checkpoint serine/threonine kinase
	CDC45	cell division cycle 45
Purple	TP53	tumor protein p53
	EGFR	epidermal growth factor receptor
	MRPS15	mitochondrial ribosomal protein S15
	PES1	pescadillo ribosomal biogenesis factor 1
	GMPS	guanine monophosphate synthase
	MARS1	Methionyl-TRNA Synthetase 1
	VAMP2	vesicle associated membrane protein 2
	ELOC	Elongin C
	POLR1B	RNA polymerase I subunit B
Tan	CTNNB1	catenin beta 1
	RBM39	RNA binding motif protein 39
	HNRNPA2B1	heterogeneous nuclear ribonucleoprotein A2/B1
	SRSF11	serine and arginine rich splicing factor 11
	FUS	FUS RNA binding protein
	RBM25	RNA binding motif protein 25
	STAT3	signal transducer and activator of transcription 3
	SNRNP70	small nuclear ribonucleoprotein U1 subunit 70
	DDX17	DEAD-box helicase 17
	RACK1	Receptor For Activated C Kinase 1
